# Supplementary material for: Modeling glioblastoma heterogeneity as a dynamic network of cell states
Source: Mol Syst Biol. 2021 Sep 16;17(9):e10105. doi: 10.15252/msb.202010105 (PMC8444284; doi:10.15252/msb.202010105)
Supplement: Supplementary file 6 — Source Data for Figure 5 [file MSB-17-e10105-s004.zip › Figure5A_sourcedata/GSEA_3017/nosigdb_stateA.GseaPreranked.1622795026485/GANDAL_AUTISM_UP_PMID29439242.html]

Details for gene set GANDAL\_AUTISM\_UP\_PMID29439242[GSEA]

|  || Dataset | state53017 |
| Phenotype | NoPhenotypeAvailable |
| Upregulated in class | na\_neg |
| GeneSet | GANDAL\_AUTISM\_UP\_PMID29439242 |
| Enrichment Score (ES) | -0.6311851 |
| Normalized Enrichment Score (NES) | -2.7370315 |
| Nominal p-value | 0.0 |
| FDR q-value | 0.0 |
| FWER p-Value | 0.0 |
Table: GSEA Results Summary

  

Fig 1: Enrichment plot: GANDAL\_AUTISM\_UP\_PMID29439242      
 Profile of the Running ES Score & Positions of GeneSet Members on the Rank Ordered List

  

| PROBE | GENE SYMBOL | GENE\_TITLE | RANK IN GENE LIST | RANK METRIC SCORE | RUNNING ES | CORE ENRICHMENT || 1 | TM4SF1 |  |  | 1 | 1.087 | 0.0589 | No |
| 2 | STC1 |  |  | 92 | 0.463 | -0.0091 | No |
| 3 | EMP3 |  |  | 101 | 0.452 | 0.0075 | No |
| 4 | SLCO4A1 |  |  | 130 | 0.420 | 0.0015 | No |
| 5 | MTHFD2 |  |  | 287 | 0.330 | -0.1424 | No |
| 6 | HSPB1 |  |  | 312 | 0.322 | -0.1496 | No |
| 7 | FKBP5 |  |  | 368 | 0.304 | -0.1900 | No |
| 8 | P4HA1 |  |  | 411 | 0.290 | -0.2177 | No |
| 9 | KIF20A |  |  | 447 | 0.281 | -0.2386 | No |
| 10 | ZFP36L1 |  |  | 532 | 0.264 | -0.3114 | No |
| 11 | PTTG1IP |  |  | 678 | -0.290 | -0.4461 | No |
| 12 | EMP1 |  |  | 724 | -0.322 | -0.4752 | No |
| 13 | IFITM2 |  |  | 864 | -0.460 | -0.5943 | No |
| 14 | ANXA2 |  |  | 872 | -0.482 | -0.5751 | No |
| 15 | SPP1 |  |  | 927 | -0.630 | -0.5964 | Yes |
| 16 | CEBPD |  |  | 939 | -0.690 | -0.5698 | Yes |
| 17 | GLIS3 |  |  | 948 | -0.767 | -0.5359 | Yes |
| 18 | IFITM3 |  |  | 954 | -0.830 | -0.4953 | Yes |
| 19 | CD44 |  |  | 957 | -0.851 | -0.4505 | Yes |
| 20 | TAGLN2 |  |  | 963 | -0.891 | -0.4066 | Yes |
| 21 | CRYAB |  |  | 967 | -0.940 | -0.3579 | Yes |
| 22 | CDKN1A |  |  | 969 | -0.987 | -0.3045 | Yes |
| 23 | ANXA1 |  |  | 972 | -1.018 | -0.2505 | Yes |
| 24 | CYR61 |  |  | 978 | -1.296 | -0.1843 | Yes |
| 25 | IGFBP5 |  |  | 983 | -1.595 | -0.1005 | Yes |
| 26 | CCL2 |  |  | 985 | -1.881 | 0.0021 | Yes |
Table: GSEA details [plain text format]

  

Fig 2: GANDAL\_AUTISM\_UP\_PMID29439242: Random ES distribution      
 Gene set null distribution of ES for **GANDAL\_AUTISM\_UP\_PMID29439242**
